# Editorial: Markers of clinical significance and treatment-seeking of psychotic experiences in non-clinical populations: potential resilience & risk factors

**DOI:** 10.3389/fpsyt.2025.1557770

**Published:** 2025-02-05

**Authors:** Suzanne Ho-wai So, Peter W. Woodruff, Salma M. Khaled

**Affiliations:** ^1^ Department of Psychology, The Chinese University of Hong Kong, Hong Kong, Hong Kong SAR, China; ^2^ School of Medicine and Population Health, The University of Sheffield, Sheffield, United Kingdom; ^3^ Department of Population Medicine, Qatar University, Doha, Qatar

**Keywords:** psychotic experiences, clinical spectrum, clinical staging, risk factors, protective factors

The continuum model posits that psychotic experiences or PEs (such as hallucinations and delusions) occur across a spectrum from diagnosis-free (i.e. non-clinical) individuals to those with a clinical diagnosis of psychosis (1, 2). PEs in non-clinical populations may appear in the forms of psychotic-like or attenuated psychotic symptoms and are more likely to do so in people at risk of psychotic disorder such as those with schizotypal traits, at-risk mental state, or psychotic prodrome (3–7). According to the clinical staging model, PEs may be either stable or progress from one stage to the next and even into full-blown clinical psychosis (8–11). However, among individuals who remain diagnosis-free, these experiences may still lead to emotional distress and functional disturbances (12–14). Therefore, it is of importance that we identify markers of clinical significance (including symptom severity, distress and impairment) and risk factors for transition of PEs into clinical psychosis for prevention and early treatment for these diverse populations (15, 16).

The last two decades of research have seen a surge in the attempts to identify (bio)markers for, and outcomes of, psychotic disorders including schizophrenia (e.g., (17–19). Despite challenges to clinical prediction models such as the heterogeneous nature of the clinical syndrome and methodological limitations (20), it has been argued that identifying markers of psychopathology is important in order to identify predisposition and characterize stages of illness (19). More recently, newer studies have begun to search for markers for the transition to psychosis based on psychometric, genetic, neurocognitive and brain imaging data (21–24). However, whereas most of these studies focused on the transition to the clinical syndrome, other aspects of clinical significance such as symptom severity, functional outcomes and emotional distress have been less studied (25).

This Special Topic, consists of five studies, all of which aimed to examine putative biological and psychosocial factors that may be of clinical significance in non-clinical individuals with PEs.

Two studies focused on risk factors that have been evident in clinical samples, and examined their associations with PEs in non-clinical samples. Gelner et al. selected one factor that represented neurodevelopmental vulnerability (attention-deficit/hyperactivity disorder, ADHD) and another factor that represented hyperarousal to environmental triggers (post-traumatic stress disorder, PTSD) (26, 27). A sizable sample (N = 3000) of young adults (aged 18-35 years) completed an online survey that assessed their PEs, ADHD, and PTSD symptoms. Based on self-reports, individuals were further categorized as ‘screened positive/negative’ on each of the two vulnerability factors. Gelner et al. found that, even after controlling for sociodemographics, PEs increased with the presence of each vulnerability factor; individuals who reported both vulnerabilities had the most PEs. The predictive power of both vulnerability factors was comparable, indicating that the risk of developing PEs is not dominated by only one vulnerability but is a product of interaction of multiple factors.

In view of the well-established speech abnormalities in psychotic patients, Olah et al. reviewed studies that examined speech features (such as semantic coherence and density, syntactic complexity, and speech connectivity) in non-clinical individuals experiencing PEs using automated analysis techniques such as Natural Language Processing, part-of-speech tagging, graph theory, and machine learning. They found that while these approaches have been used in non-clinical samples at various stages of the psychotic continuum, the number of studies were too small to allow un-biased estimates in light of the lack of representative samples, standardized methods, and longitudinal observations. This review highlighted automated speech analysis as a promising approach in capturing subtle speech abnormalities in non-clinical samples (avoiding confounding factors in clinical samples such as cognitive decline and medication effects), thus advocating for more research in this area.

Tuin et al. focused on a putative protective factor, namely positive affect. Individuals at risk for psychosis (N = 96) were categorized into four subgroups defined according to the clinical staging model. Analysis of their daily diary data over 90 days revealed a significant, within-day, bi-directional association between positive affect and PEs, where a higher level of positive affect was associated with a lower level of PEs and vice versa. While previous studies focused on ameliorating risk factors, this paper suggested that improving positive affect may be an important early intervention approach for PEs, especially in those at ultra-high risk for psychosis.

As both cannabis use and belief updating under uncertainty has been associated with psychosis in previous studies, Liang et al. compared ‘belief updating under uncertainty’ between 49 regular cannabis users and 52 non-cannabis users. Rather than treating PEs as in the above studies, the main analysis in Liang et al. concerned the association between cannabis use and ‘belief updating under uncertainty’ measured by the Space Task. Even though both cannabis users and non-users were not different in their performance on this task, cannabis users exhibited a higher level of delusional ideation but a comparable level of PEs (except for cognitive perceptual deficits) compared with the non-cannabis users. While frequency of cannabis use was associated with worse performance on belief updating, caution should be exercised to avoid directly inferring its association with PEs.

Lastly, with a sample of 752 patients in the maintenance-phase of schizophrenia, Yuan et al. examined uric acid as a blood biomarker of cognitive function. In view of evidence of a negative association between uric acid and risks to multiple neurodegenerative diseases, Yuan et al. compared patients with low-normal, middle-normal, high-normal, and high levels of uric acid on their Mini Mental State Examination performance. After adjusting for age, sex, body mass index, history of smoking and drinking, general health indicators, antipsychotic dosage and schizophrenia symptoms, there was a significant and positive association between level of uric acid and cognitive function (orientation, immediate memory, delayed recall, and languages). Potential neuroprotective properties of uric acid are discussed.

Overall, the present Research Topic underlines multiple approaches of evaluating biological, psychosocial, and environmental markers of clinical significance for individuals at various stages of the psychosis continuum paving the way for more transdiagnostic approaches to prevention and treatment of these heterogenous conditions.
